# Relationship Between the Axis of the Humeral Heads and the Spinal Sagittal Alignment in Asymptomatic Volunteers

**DOI:** 10.7759/cureus.71587

**Published:** 2024-10-16

**Authors:** Ikuho Yonezawa, Makoto Yoshida, Tomohiro Shinozaki, Masatake Ino, Koji Yamada, Yusuke Nakao, Yasuo Oohori

**Affiliations:** 1 Orthopedic Surgery, Tokyo Kamata Hospital, Tokyo, JPN; 2 Orthopedic Surgery, Sangubashi Spine Surgery Hospital, Tokyo, JPN; 3 Information and Computer Technology, Faculty of Engineering, Tokyo University of Science, Tokyo, JPN; 4 Orthopedic Surgery, Gunma Spine Center, Takasaki, JPN; 5 Orthopedic Surgery, Nakanojima Orthopedic Clinic, Tokyo, JPN

**Keywords:** humeral head axis, lumbar lordosis, pelvic incidence, pelvic tilt, shoulder incidence, shoulder pelvic angle, shoulder tilt, spinal sagittal alignment, t1 slope, thoracic kyphosis

## Abstract

Background

Currently, there is no known correlation between relevant parameters of respective non-adjacent spinal segments, which suggests the possibility of a third external factor. To our knowledge, no study has examined the humeral head position in consideration of spinal sagittal alignment.This study aims to establish new parameters related to the humeral head axis and evaluate the relationship between the axis of the humeral head and spinal sagittal alignment in asymptomatic volunteers.

Methods

Standing posteroanterior and lateral radiographs of the entire spine were obtained from 62 asymptomatic volunteers. We analyzed the relationship of the newly established parameters related to the humeral head axis We analyzed the relationship of the newly established parameters related to the humeral head axis and other established spinal parameters. The new parameters are: shoulder tilt (ST: the angle between a line drawn from the center of the humeral head to the center of the superior endplate of T1 and vertical line through the center of the humeral head), shoulder incidence (SI: the angle between a line perpendicular to the upper endplate of the T1 vertebra and a line joining the center of the upper endplate of the T1 vertebra and the axis of the humeral head), shoulder pelvic angle (SPA: the angle between a line joining the axis of the humeral head and the femoral head and a line joining the axis of the femoral heads and the center of the upper endplate of the S1 vertebra), C2 shoulder angle (C2SA: the angle between a line joining the C2 body center and the axis of the humeral head and a line joining the axis of the humeral head and the center of the upper endplate of the T1 vertebra). Statistical analysis was performed using Pearson’s correlation coefficient.

Results

Mean ST, SI, SPA, and C2SA were 20.0±20.6°, 43.7±21.6°, 7.7±6.6°, and 17.8±13.5° respectively. The SI was found to be correlated with pelvic tilt (r=0.373), lumbar lordosis (r=0.351), and thoracic kyphosis (r=0.469). Pelvic incidence was correlated with SPA (r=0.724), T1 pelvic angle (r=0.691), and C2 pelvic angle (r=0.667). Correlations were observed between SPA and T1PA (r=0.955) and C2PA (r=0.956), and between TIPA and C2PA (r=0.985).

Conclusions

This report introduced novel parameters related to humeral head position and confirmed their correlation with sagittal alignment from the cervical spine to the pelvis. Humeral head position may play a role in maintaining spinal sagittal balance.

## Introduction

A chain of correlations is known to exist among the relevant parameters of respective spinal segments, such as between pelvic incidence (PI) and lumbar lordosis (LL), LL and thoracic kyphosis (TK), and TK and cervical lordosis (CL) [1-5]. However, a number of serious questions remain unresolved. With respect to the cervical sagittal alignment, a correlation was confirmed between CL and T1 slope, and T1 slope and TK [6,7], but not with lumbopelvic alignment, therein suggesting the possibility of a third unknown external factor at work.

The humeral head forms the shoulder joint with the glenoid cavity of the scapula, which moves in conjunction with the humeral head. The levator scapulae and trapezius muscles arise from the cervical spine and are inserted into the scapula; the contraction of these muscles assists in cervical spine extension [8]. Thus, we postulated that the humeral head position might have an indirect effect on the cervical alignment through the scapula and connecting muscles.

The current report was formulated to establish new parameters related to the humeral head axis and evaluate the influence of the position of the humeral head on overall sagittal alignment.

## Materials and methods

Study population

A cohort of 62 Japanese asymptomatic adult volunteers was analyzed. All volunteers signed written informed consent. The exclusion criteria were as follows: 1) neck and back pain, 2) radiculopathy, 3) neurological deficit, and 4) a history of diagnosis and treatment related to the spine. This study was approved by the institutional review board, Sangubashi Spine Surgery Hospital, Tokyo (Approved code: N0102).

Data collection and radiographic measurement

Demographics and clinical characteristics including age, sex, and body mass index (BMI), and all spinopelvic-related radiographic parameters were collected prospectively.

Standing posteroanterior and lateral radiographs of the entire spine were obtained using the TOSHIBA Medical Systems BLR-15AA, Tokyo, Japan. Volunteers were asked to stand with extended knees. The elbows were flexed with knuckles in the supraclavicular fossa bilaterally. Eyes were strictly oriented straight forward using a mirror adapted to the patient’s height. Images were obtained from the skull to the proximal femur. All images were taken by two trained radiographers with more than 15 years of experience in performing whole-spine radiographs.

**Figure 1:**
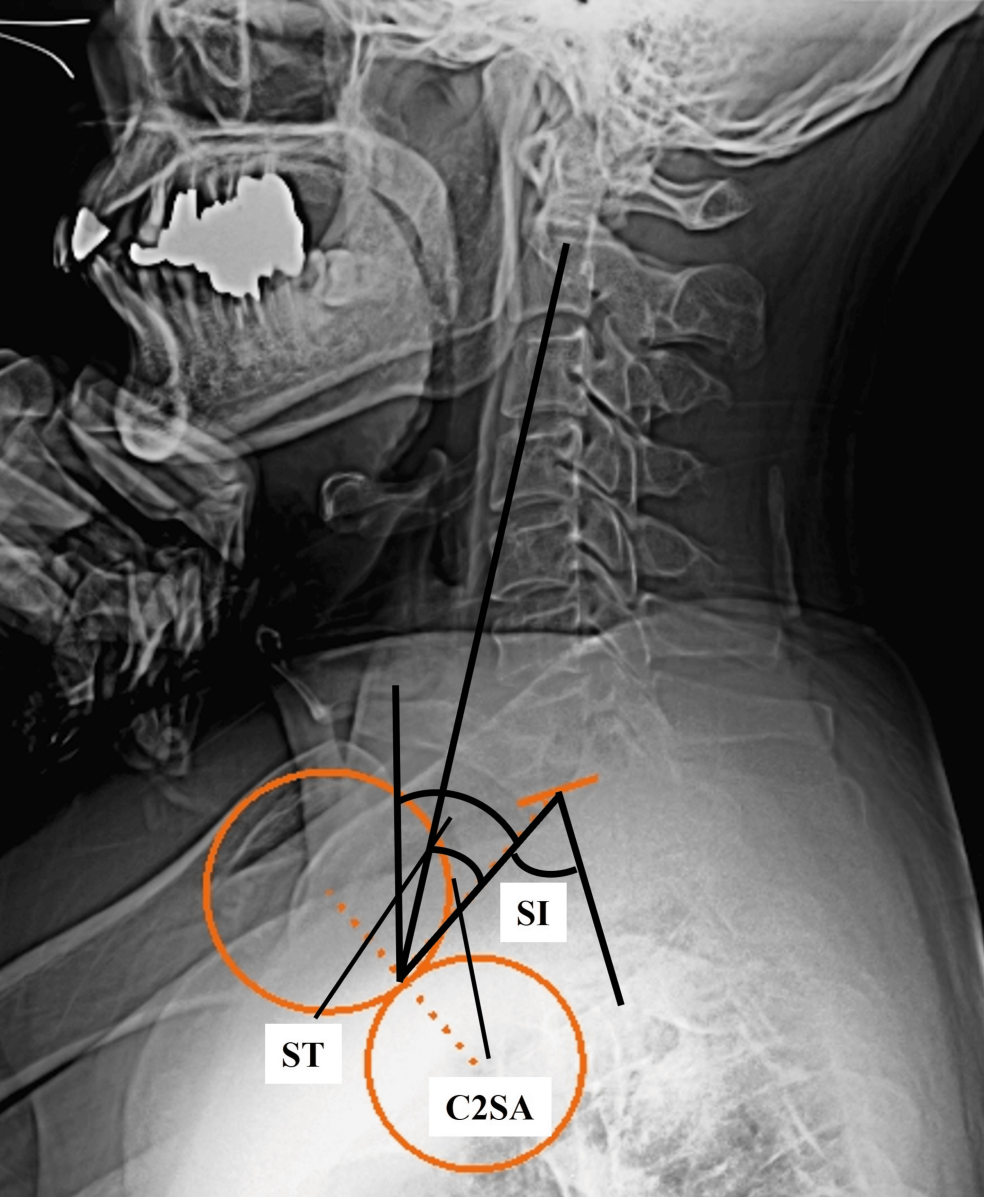
Examples of new parameters; ST, SI, and C2SA. Parameters: ST, the angle between the line joining the center of the upper endplate of the T1 vertebra and the axis of the humeral head and the vertical axis; SI, the angle between a line perpendicular to the upper endplate of the T1 vertebra and a line joining the center of the upper endplate of the T1 vertebra and the axis of the humeral head; C2SA, the angle between the line joining the C2 body center and axis of the humeral head and the line joining the axis of the humeral head and the center of the upper endplate of the T1 vertebra. ST, shoulder tilt; SI, shoulder incidence; C2SA, C2 pelvic angle.

The axis of the humeral head-related parameters measured included shoulder tilt (ST: the angle between a line drawn from the center of the humeral head to the center of the superior endplate of T1 and a vertical line through the center of the humeral head), shoulder incidence (SI: the angle between a line perpendicular to the upper endplate of the T1 vertebra and a line joining the center of the upper endplate of the T1 vertebra and the axis of the humeral head), shoulder pelvic angle (SPA: the angle between a line joining the axis of the humeral head and the femoral head and a line joining the axis of the femoral heads and the center of the upper endplate of the S1 vertebra), and C2 shoulder angle (C2SA: the angle between a line joining the C2 body center and the axis of the humeral head and a line joining the axis of the humeral head and the center of the upper endplate of the T1 vertebra). The formula, “SI=ST+T1 slope” is comparable to the known “PI=PT+SS.” Examples of the new parameters are shown in Figure 1. In the case of the C2SA, the angles opening to the right were considered positive.

Pelvic and spinal parameters measured included pelvic tilt (PT: the angle between the line joining the center of the sacral endplate and the axis of the femoral head and the vertical axis), pelvic incidence (PI: the angle between a line perpendicular to the sacral endplate and a line joining the center of the sacral plate and the axis of the femoral head), lumbar lordosis (LL: the angle between the upper endplate of the L1 vertebra and the upper endplate of the S1 vertebra), T1-12 kyphosis (TK: the angle between the upper endplate of the T1 vertebra and the lower endplate of the T12 vertebra), cervical lordosis (CL: the angle between the upper endplate of the C2 vertebra and the lower endplate of the C7 vertebra), T1 slope (the angle between a horizontal line and superior endplate of the T1 vertebra), C2 pelvic angle (C2PA: the angle between a line joining the C2 body center and a line joining the axis of the femoral head and the center of the upper endplate of the S1 vertebra), and T1 pelvic angle (T1PA: the angle between a line joining the T1 body center and a line joining the axis of the femoral head and the center of the upper endplate of the S1 vertebra).

Statistical analysis

All radiographs were acquired in digital format. The parameters described above were then measured using Surgimap, version 2.3.2.1 (Spine Software, New York, NY, USA). Statistical analysis was performed using Pearson’s correlation coefficient. Statistical significance was considered at p < 0.01.

To assess the reliability of the new parameters, two observers measured the ST, SI, SPA, and C2SA in 10 sample cases randomly selected from 62 asymptomatic volunteers on two separate occasions at least 72 hours apart. The observers were two spine surgeons with 11 and 29 years of experience, respectively, in spine measurements. The average of both spine surgeons’ measurements was used for the analysis. Inter-observer and intra-observer reliabilities were assessed using the intraclass correlation coefficient (ICC). The internal consistency of measurements was characterized as excellent (ICC≥0.9), good (0.7≤ICC<0.9), acceptable (0.6<ICC≤0.7), poor (0.5≤ICC<0.6), or unpredictable (ICC<0.5) [9]. Statistical analyses were performed using the statistical software IBM SPSS Statistics for Windows, version 26 (IBM Corp., Armonk, NY, USA).

## Results

A total of 62 volunteers (43 males, 19 females) were analyzed. The average age was 39.3±10.4 years (range 23-64). The average BMI was 23.0±3.9 kg/m2 (Table 1).

**Table 1:** Baseline characteristics and radiographic parameters. PT, pelvic tilt; PI, pelvic incidence; LL, lumbar lordosis; SS, sacral slope; TK, thoracic kyphosis; CL, cervical lordosis; ST, shoulder tilt; SI, shoulder incidence; C2SA, C2 shoulder angle; SPA, shoulder pelvic angle; T1PA, T1 pelvic angle, C2PA, C2 pelvic angle.

	Mean	SD	Minimum	Maximum
BMI (kg/m^2^)	23.0	3.9	17.2	37.5
Age (y.o)	39.3	10.4	23.0	64.0
PT (°)	8.8	7.4	-8.0	23.0
PI (°)	51.1	10.1	30.0	76.0
LL (°)	53.0	9.4	30.0	69.0
SS (°)	42.3	7.8	26.0	57.0
PI-LL (°)	-1.9	7.8	-26.0	10.0
TK (°)	40.3	11.3	12.0	66.0
CL (°)	4.3	11.9	-26.0	25.0
T1 slope (°)	23.7	7.5	7.0	41.0
ST (°)	20.0	20.6	-39.0	61.0
SI (°)	43.7	21.6	-32.0	78.0
C2SA (°)	17.8	13.5	-20.1	43.2
SPA (°)	7.7	6.6	-8.9	19.8
T1PA (°)	5.9	5.9	-10.2	18.5
C2PA (°)	7.4	6.2	-10.5	20.4

Radiographic results

Measurement of Parameter

The parameters related to the axis of the humeral head were as follows: ST (20.0±20.6°), SI (43.7±21.6°), SPA (7.7±6.6°), and C2SA (17.8±13.5°). The mean Cobb angles were as follows: LL (53.0±9.4°), TK (40.3±11.3°), and CL (4.3±11.9°). The pelvic related parameters were as follows: PI (51.1±10.1°), PT (8.8±7.4°), PI-LL (-1.9±7.8°), T1 slope (23.7±7.5°), C2PA (7.4±6.2°), and T1PA (5.9±5.9°) (Table 1).

Inter- and Intra-observer Reliability for New Radiographic Parameters

Excellent interobserver reliability was observed for all the new radiographic parameters. Intra-observer reliability was good to excellent for all new variables (Table 2).

**Table 2:** Inter- and intra-observer reliability for new radiographic parameters. ICC, intraclass correlation coefficient.

	Inter-rater ICC	Inter-rater reliability	Intra-rater ICC	Intra-rater reliability
Shoulder tilt	0.988	Exellent	0.929	Exellent
Shoulder incidence	0.934	Exellent	0.832	Good
Shoulder pelvic angle	0.987	Exellent	0.939	Exellent
C2 shoulder angle	0.982	Exellent	0.880	Good

Correlations for Parameters

Correlations between parameters are shown in Table 3. The SI correlated with lumbosacral alignment (PT, r=0.373; LL, r=0.351) and TK (r=0.469), indicating the influence of humeral head position on overall sagittal alignment. PI correlated with SPA (r=0.724), T1PA (r=0.691), and C2PA (r=0.667), demonstrating the influence of pelvic morphology on the location of the humeral head and T1 and C2 vertebrae. Correlations were observed between SPA and T1PA (r=0.955) and C2PA (r=0.956), and between TIPA and C2PA (r=0.985). These results indicate a reciprocal coordinative relationship between the positions of the humeral head, T1 vertebra, and C2 vertebra.

**Table 3:** Correlation for parameters in asymptomatic volunteers. PT, pelvic tilt; PI, pelvic incidence; LL, lumbar lordosis; SS, sacral slope; TK, thoracic kyphosis; CL, cervical lordosis; ST, shoulder tilt; SI, shoulder incidence; C2SA, C2 shoulder angle; SPA, shoulder pelvic angle; T1PA, T1 pelvic angle; C2PA, C2 pelvic angle. * Correlation is significant at the 0.05 level (2-tailed); ** Correlation is significant at the 0.01 level (2-tailed).

	BMI	Age	PT	PI	LL	SS	PI-LL	TK	CL	T1 slope	ST	SI	C2SA	SPA	T1PA	C2PA
BMI	1															
Age	0.235	1														
PT	-0.122	0.009	1													
PI	-0.105	0.236	0.659^**^	1												
LL	-0.122	0.239	0.208	0.679^**^	1											
SS	-0.021	0.315^*^	-0.124	0.652^**^	0.690^**^	1										
PI-LL	0.009	0.018	0.602^**^	0.483^**^	-0.315	0.021	1									
TK	-0.005	0.242	0.211	0.194	0.485^**^	0.046	-0.329	1								
CL	0.065	0.279^*^	-0.170	0.045	0.148	0.238	-0.119	0.437^**^	1							
T1 slope	0.064	0.305^*^	-0.018	0.075	0.156	0.106	-0.088	0.804^**^	0.595^**^	1						
ST	0.170	-0.040	0.398^**^	0.272^*^	0.312^*^	-0.026	-0.016	0.199	-0.047	-0.058	1					
SI	0.183	0.069	0.373^**^	0.287^*^	0.351^**^	0.012	-0.045	0.469^**^	0.163	0.292^*^	0.938^**^	1				
C2SA	0.230	0.050	0.427^**^	0.314^*^	0.336^**^	-0.008	0.008	0.304^*^	-0.052	0.075	0.913^**^	0.900^**^	1			
SPA	-0.015	0.142	0.903^**^	0.724^**^	0.194	0.044	0.704^**^	0.194	-0.059	0.146	0.398^**^	0.431^**^	0.459^**^	1		
T1PA	-0.010	0.168	0.878^**^	0.691^**^	0.097	0.019	0.776^**^	0.133	-0.098	0.128	0.166	0.202	0.263^*^	0.955^**^	1	
C2PA	0.016	0.173	0.895^**^	0.667^**^	0.108	-0.028	0.731^**^	0.215	-0.119	0.180	0.210	0.263^*^	0.336^**^	0.956^**^	0.985^**^	1

## Discussion

The present study examined the potential influence of the humeral head in relation to the overall and cervical alignment by establishing new parameters: ST, SI, SPA and C2SA. C2SA simultaneously accounts for both cervical inclination and T1 vertebral retroversion, similar to the T1 pelvic angle [10].

From the local anatomy, we hypothesized the linkage of the humeral head to the cervical sagittal alignment through the scapula and connecting muscles such as the levator scapulae and trapezius [8]. The results yielded favorable outcomes, elucidating a network of interrelations among the relevant spinal parameters.

**Figure 2:**
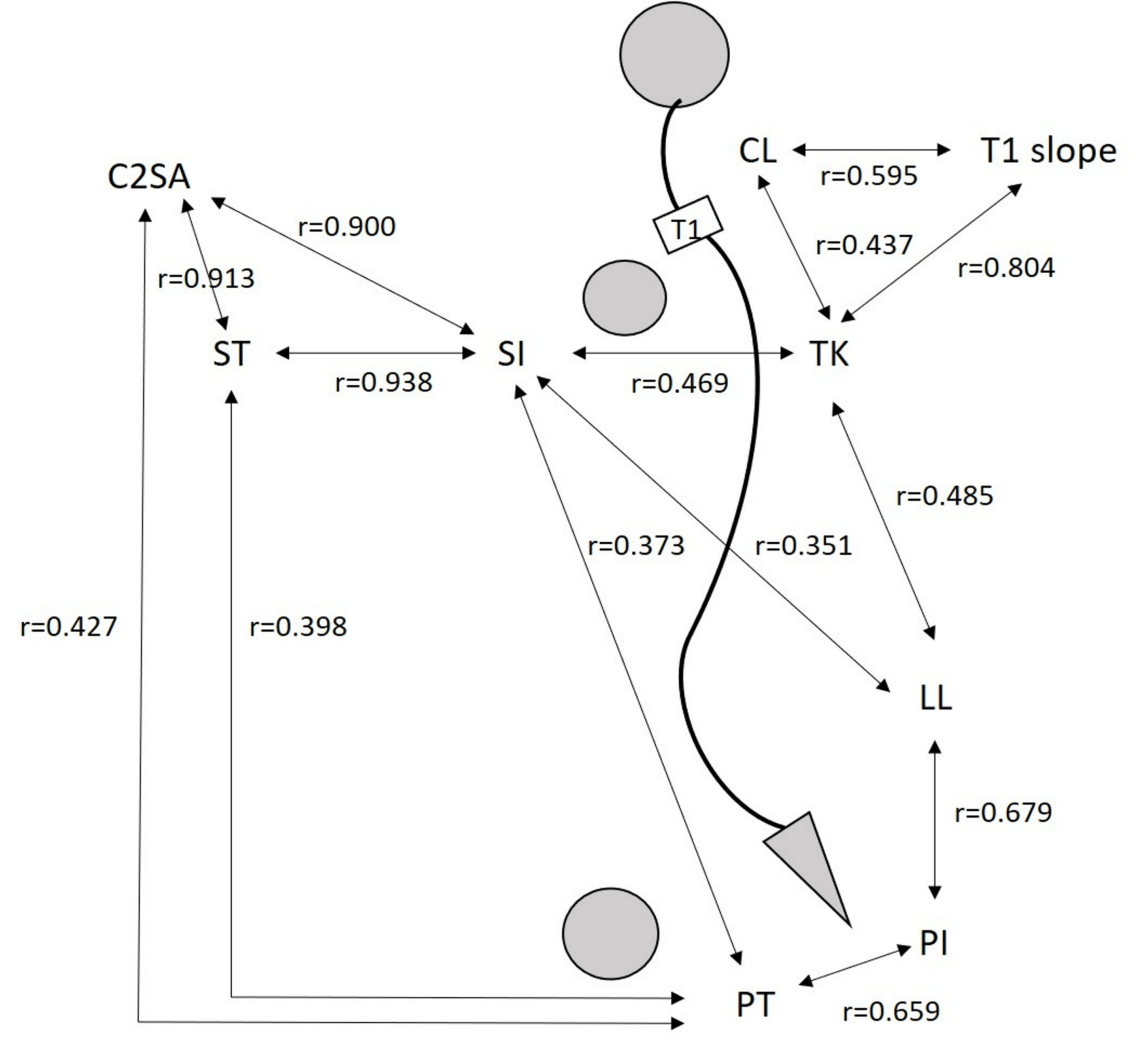
Chain of correlation linking cervical alignment to the pelvis, lumbar and thoracic spine in asymptomatic volunteers. Image credit: Ikuho Yonezawa.

**Figure 3:**
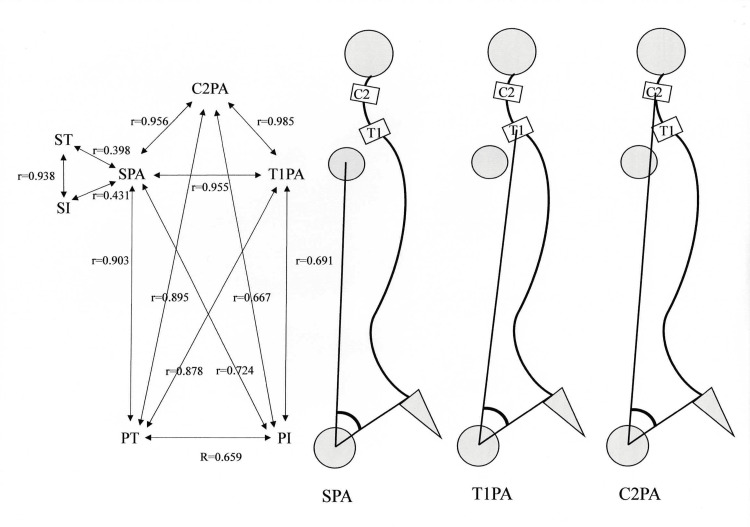
Correlation linking SPA, T1PA and C2PA to the pelvis in asymptomatic volunteers. SPA, shoulder pelvic angle; T1PA, T1 pelvic angle; C2PA, C2 pelvic angle. Image credit: Ikuho Yonezawa.

Correlations of SI versus PT, LL, and TK indicate the significant link of the humeral head position to each spinal segmental alignment. The strong correlations of PI with SPA, T1PA, and C2PA, are particularly noteworthy as the correlations demonstrate that pelvic morphology influences the relative locations of the humeral head, T1, and C2 vertebrae (Figure 2). Strong correlations among SPA, T1PA, and C2PA attest to the reciprocal coordinative mechanism among the humeral head, T1, and C2 vertebrae (Figure 3).

We found that the inter-observer and intra-observer reliability of the ST, SI, SPA, and C2SA was good to excellent, respectively. Compared to the femoral head, identifying the rim of the humeral head on lateral radiographs is somewhat difficult due to a lack of clarity. However, digital radiography provides reliable measurements by adjusting the contrast.

Previous studies on asymptomatic patients have established correlations between PI and LL, LL and TK, TK and CL, CL and T1 slope, T1 slope, and TK, and finally, C2SVA and T1 slope [1-7,11], indicating the existence of exquisite networks of collaboration among the respective spinal segments.

With respect to cervical sagittal alignment in asymptomatic volunteers, Lee et al. introduced a new concept to assess the influence of the thoracic inlet on cervical sagittal alignment and established the correlations between Thoracic inlet angle (TIA) and T1 slope and between T1 slope and CL [7]. However, the correlation of TIA did not extend to other spinal segments.

The present study using asymptomatic volunteers also ascertained the correlations between PI and LL, LL and TK, TK and CL, T1 slope and CL, and TK. However, the correlations were still confined to the two adjacent spinal segments, such as lumbar lordosis and thoracic kyphosis, while no significant correlations were found among non-adjacent spinal segments such as lumbar and cervical lordosis. Nonetheless, the present study demonstrated correlations among the parameters of the respective non-adjacent segments, such as between PT versus SI, ST, and C2SA.

This study has some limitations. First, samples were insufficient for analysis beyond the present studies, particularly for age- and sex-specific analyses. Second, this study was based on asymptomatic Japanese volunteers. Therefore, further large-scale multi-center studies assessing large numbers of individuals from different ethnic populations are warranted.

## Conclusions

This study introduced novel parameters related to humeral head position and confirmed their correlation with sagittal spinal alignment from the cervical spine to the pelvis. The humeral head position may play an indispensable role in maintaining spinal sagittal balance. We hope that the analysis of the humeral head position and related parameters will provide a new perspective toward understanding the overall spinal sagittal alignment.
